# Economic evaluations of artificial intelligence-based healthcare interventions: a systematic literature review of best practices in their conduct and reporting

**DOI:** 10.3389/fphar.2023.1220950

**Published:** 2023-08-08

**Authors:** Jai Vithlani, Claire Hawksworth, Jamie Elvidge, Lynda Ayiku, Dalia Dawoud

**Affiliations:** ^1^ National Institute for Health and Care Excellence, London, United Kingdom; ^2^ National Institute for Health and Care Excellence, Manchester, United Kingdom; ^3^ Faculty of Pharmacy, Cairo University, Cairo, Egypt

**Keywords:** artificial intelligence, cost effectiveness, cost utility, simulation models, health economic evaluation, mixed-methods, systematic review

## Abstract

**Objectives:** Health economic evaluations (HEEs) help healthcare decision makers understand the value of new technologies. Artificial intelligence (AI) is increasingly being used in healthcare interventions. We sought to review the conduct and reporting of published HEEs for AI-based health interventions.

**Methods:** We conducted a systematic literature review with a 15-month search window (April 2021 to June 2022) on 17^th^ June 2022 to identify HEEs of AI health interventions and update a previous review. Records were identified from 3 databases (Medline, Embase, and Cochrane Central). Two reviewers screened papers against predefined study selection criteria. Data were extracted from included studies using prespecified data extraction tables. Included studies were quality assessed using the National Institute for Health and Care Excellence (NICE) checklist. Results were synthesized narratively.

**Results:** A total of 21 studies were included. The most common type of AI intervention was automated image analysis (9/21, 43%) mainly used for screening or diagnosis in general medicine and oncology. Nearly all were cost-utility (10/21, 48%) or cost-effectiveness analyses (8/21, 38%) that took a healthcare system or payer perspective. Decision-analytic models were used in 16/21 (76%) studies, mostly Markov models and decision trees. Three (3/16, 19%) used a short-term decision tree followed by a longer-term Markov component. Thirteen studies (13/21, 62%) reported the AI intervention to be cost effective or dominant. Limitations tended to result from the input data, authorship conflicts of interest, and a lack of transparent reporting, especially regarding the AI nature of the intervention.

**Conclusion:** Published HEEs of AI-based health interventions are rapidly increasing in number. Despite the potentially innovative nature of AI, most have used traditional methods like Markov models or decision trees. Most attempted to assess the impact on quality of life to present the cost per QALY gained. However, studies have not been comprehensively reported. Specific reporting standards for the economic evaluation of AI interventions would help improve transparency and promote their usefulness for decision making. This is fundamental for reimbursement decisions, which in turn will generate the necessary data to develop flexible models better suited to capturing the potentially dynamic nature of AI interventions.

## 1 Introduction

The use of artificial intelligence (AI) has significantly grown in the healthcare sector. Exploiting its ability to streamline tasks, provide real-time analytics, and process larger quantities of data has contributed to its increased prominence (Panch et al., 2018). Additionally, it may have the potential to deliver quality care at lower costs. AI is being used to address challenges ranging from staff shortages to ageing populations and rising costs (Dall et al., 2013). The number of AI technologies approved by the US Food and Drink Administration (FDA) was nearly 350 between 2016 and mid-2021, compared to less than 30 in the preceding 19 years (Miller, 2021).

Several systematic reviews have been published that examine health economic evaluations (HEEs) for AI in healthcare. The most recent is Voets et al. (1 April 2021) (Voets et al., 2022), who searched for publications from 5 years prior and included 20 full texts, discussing the methods, reporting quality and challenges. They found that automated medical image analysis was the most common type of AI technology, just under half of studies reported a model-based HEE, and the reporting quality was moderate. Overall, Voets et al. concluded that HEEs of AI in healthcare often focus on costs rather than health impact, and insight into benefits is lagging behind the technological developments of AI.

An up-to-date representation of the economic evidence base may be insightful. Clearly, AI is a rapidly developing area in healthcare, demonstrated by the National Institute for Health and Care Excellence (NICE) recently incorporating AI technologies into its Evidence Standards Framework (Unsworth et al., 2021; National Institute for Health and Care Excellence, 2022). While some of this rise may be attributable to changes in legislation, it indicates the importance of AI in the current healthcare climate and the need to have a contemporary understanding of its economic value. Additionally, the COVID-19 pandemic has led to a rapid increase in the digitalization of data and health services including teleconsultations, online prescriptions and remote monitoring (Gunasekeran et al., 2021). Therefore, we sought to update the Voets et al. systematic review. We report updated results consistent with the original review, by disaggregating the HEEs into costs, clinical effectiveness, modelling characteristics and methodologies to understand common techniques, limitations, assumptions, and uncertainties. This update allows us to advance the discussion around whether existing modelling methods and reporting standards are suitable to appropriately assess the cost effectiveness of AI technologies compared to non-AI technologies in healthcare.

This review was undertaken to inform ongoing work within the HTx project. HTx is a Horizon 2020 project supported by the European Union lasting for 5 years from January 2019. The main aim of HTx is to create a framework for the Next-Generation Health Technology Assessment (HTA) to support patient-centred, societally oriented, real-time decision-making on access to and reimbursement for health technologies throughout Europe.

## 2 Data and methods

### 2.1 Literature search strategy

The search strategy included the period from 1 April 2021 to 17 June 2022, in order to update the original search conducted by Voets et al. (Voets et al., 2022). The original search used the PubMed and Scopus databases. For the present update, the original search strategy was translated for use in MEDLINE, EMBASE, via the Ovid platform, and Cochrane Central, via Wiley. These databases were preferred due to their accessibility, and searching all 3 was considered to provide comparable coverage to PubMed and Scopus (Ramlal et al., 2021).

The search strategy was simplified into 2 concept pathways: 1. “Artificial intelligence” and 2. “Health economic evaluations”. The search queries in Supplementary Appendix SA show the strategies divided into their respective databases. Subsequent terms in the AI pathway included, “artificial intelligence”, “machine learning”, and “data driven”. The second pathway included terms such as, “cost effectiveness”, “health outcomes”, “cost”, “budget”. An English language query was applied to the search strategy. The initial database selection and search strategies were guided by NICE information specialists. The review and search protocol were not registered.

### 2.2 Inclusion and exclusion criteria

Studies were included if they were a HEE of an AI intervention and a comparator, such as current standard of care or a non-AI intervention. This included trial-based economic evaluations and model-based studies. There were no exclusion criteria on types of economic evaluation, such that cost-effectiveness analyses (CEAs), cost-utility analyses (CUAs), cost-minimization analyses (CMA) and budget impact analyses (BIAs) were included. We term all of these as HEEs, which are defined as the “comparative analysis of alternative courses of action in terms of both their costs and consequences” (Rudmik and Drummond, 2013). CEAs evaluate whether an intervention provides relative value, in terms of cost and health outcomes, to a respective comparator. CUAs are a subset of CEAs where the health outcome includes a preference-based measure such as the Quality Adjusted Life Year (QALY). BIA studies evaluate the affordability of an intervention for payers to allocate resources. Included studies reported a quantitative health economic outcome such as costs, or costs in relation to effectiveness. For the exclusion criteria in the initial screening of titles and abstracts, studies that were not original research or systematic reviews such as commentaries, letters, and editorials were excluded. Overall, the inclusion and exclusion criteria were consistent with Voets et al. (Voets et al., 2022).

After duplicates were removed, 2 reviewers independently screened titles, and abstracts. The reviewers discussed any discrepancies, and where agreement could not be reached, an independent third reviewer was consulted. The same process was followed for subsequent full-text screening.

### 2.3 Data extraction

The data extraction was initially completed by 1 reviewer, and then validated by a second reviewer who independently extracted and compared data from the included studies. The extraction strategy was divided into three components, the first and second components included the characteristics and the methodological details of the studies. The former included aspects such as the purpose of the AI technology, medical field, funding, care pathway phase (prevention, diagnostics, monitoring, treatment) and the type of AI (i.e., pattern recognition, risk prediction, etc.). The second table of methodological details included aspects such as the type of HEE, the comparator, and the outcome measure. The third component was relevant only for model-based HEEs, extracting parameters such as model states, time horizon, and details of sensitivity analyses.

### 2.4 Data analysis

The extracted data were synthesised using a narrative approach as heterogeneity between studies inhibited the utility of a quantitative synthesis. Descriptive statistics were used to summarize the characteristics of the retrieved studies, where appropriate.

### 2.5 Quality assessment

The quality assessment of all included studies was conducted using the NICE quality appraisal checklist for economic evaluations (National Institute for Health and Care Excellence, 2012). This checklist has been adopted in the literature of economic evaluation reviews (Elvidge et al., 2022) and is used by NICE when assessing HEE evidence for all public health guidelines. Included studies with a decision-analytic model were quality assessed independently by 2 reviewers using the methodological checklist section of the quality appraisal checklist. The checklist has 11 individual questions to create an overall assessment of whether there are minor-, potentially serious-, or very serious limitations that affects the robustness of the results. Quality assessment was not used as part of the exclusion criteria, as one of the research aims was to explore the reporting standards.

Although it is not possible to fully remove the potential of bias due to the subjective nature of the assessment, pre-set criteria were created to minimize its effects. The criteria are as follows: studies with very serious limitations included studies that had significant modelling discrepancies that could materially change the cost-effectiveness conclusion (e.g., the intervention changing from dominant to dominated). Also, very serious limitations are derived from a financial conflict of interest, where the developer of the AI technology also funded the HEE. Potentially serious limitations refer to methodological uncertainties which may change the quantitative result (e.g., an increase in the cost-effectiveness ratio), however the outcome could stay the same (e.g., the increase is not meaningful). All other limitations were considered to be minor limitations. The reviewers discussed any discrepancies in their quality assessments, and if major disagreements emerged, an independent third reviewer was consulted.

## 3 Results

### 3.1 Search results

**FIGURE 1:**
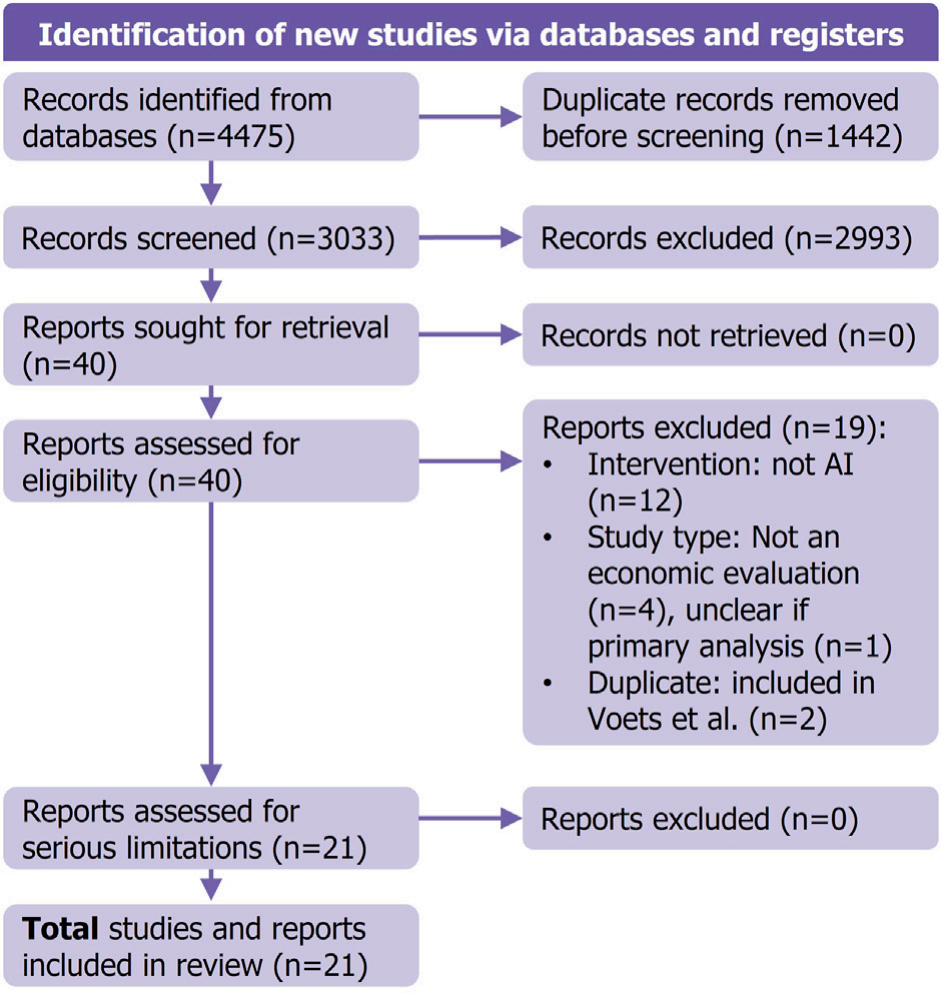
PRISMA flowchart describing study selection and reasons for exclusion during full-text screening.

The searches across the 3 databases yielded 4,475 records, resulting in 3,033 unique records following deduplication (Table 1). After screening titles and abstracts against the study selection criteria 2,993 were excluded due to not relating to a human health intervention, not reporting a HEE, not relating to an AI-based intervention, or being a excludable study type (e.g., commentary). Therefore, 40 studies proceeded to full-text screening. Of those, 16 were excluded based on the selection criteria, and 2 were excluded as duplicates that had already been included in the Voets et al. review (Voets et al., 2022). We excluded a further study due to unclear reporting about whether it was a primary analysis or a review of other economic models. Therefore, 21 studies remained which were suitable for data extraction. See Figure 1 for the PRISMA flowchart showing the inclusion and exclusion stages.

**TABLE 1:** Database search results.

Databases	Date searched	Database version	Number of records retrieved
Medline (Ovid)	17th June 2022	Ovid MEDLINE(R) ALL <1946 to 16 June 2022>	1,876
Embase (Ovid)	17th June 2022	Embase <1974 to 2022 June 16>	2,529
Cochrane Central (Wiley)	17th June 2022	Issue 5 of 12, May 2022	70
			4,475

### 3.2 Overview of included studies

The general characteristics of the 21 included studies are presented in Table 2. The majority were published in 2022. There was a wide variation of AI interventions in different medical fields. The most frequent were general medicine and oncology (each 4/21, 19%), followed by ophthalmology and respiratory medicine (each 3/21, 14%), cardiology (2/21, 10%), and dermatology, mental health, radiology, sleep and analgesics (each 1/21, 5%). The interventions spanned the screening (9/21, 43%), diagnosis (8/21, 38%), treatment (1/21, 5%) and monitoring (3/21, 14%) stages of the clinical pathway. The most common type of AI evaluated was automated image analysis (9/21, 43%). Others were risk prediction (6/21, 29%), pattern recognition (2/21, 10%), personalized treatment recommendation (1/21, 5%), clinical decision support (1/21, 5%) and combined risk prediction and clinical decision support (2/21, 10%). Most studies were funded by governments and industry (each 5/21, 24%), followed by academia (3/21, 14%). Two (2/21, 10%) were jointly funded by industry and academia and one (1/21, 5%) was funded by the European Commission.

**TABLE 2:** Characteristics of the included studies.

Main author	Year	Population	Location	Description of AI intervention	Medical field	Care pathway phase	AI technology	Funding
Adams et al. (2021)	2021	A representative cohort of 3,197 baseline screening patients	United States	Risk score predictor	Oncology	Screening	Risk prediction	Industry
Areia et al. (2022)	2022	A hypothetical cohort of 100,000 individuals aged 50–100 years	United States	AI tools to detect precancerous polyps during colonoscopy	Oncology	Screening	Pattern recognition	EU Commission and JSPS
de Vos et al. (2022)	2022	Dutch Patients	Holland	Decision-making support tool to discharge patients from ICU	General	Diagnostic	Clinical decision support	None
Delgadillo et al. (2022)	2022	Patients with common mental health disorders	United Kingdom	Decision-support tool providing personalized treatment recommendations (stratified care)	Mental health	Treatment	Personalised treatment recommendation	Industry and Academia
Ericson et al. (2022)	2022	Adult patients who were not diagnosed with sepsis at the time of admission	Sweden	Early detection of sepsis	General	Diagnostic	Risk prediction	Industry
Fusfeld et al. (2022)	2022	Kidney transplant recipients receiving a for-cause biopsy	United States	MMDx-Kidney assesses the probability of biopsy rejection or injury	General	Diagnostic	Pattern recognition	Industry
Huang et al. (2022)	2022	Diabetes patients without retinopathy	Rural China areas	Automated retinal image analysis system for diabetic retinopathy screening	Ophthalmology	Screening	Automated image analysis	Industry and Academia
Kessler et al. (2021)	2021	High-risk Medicaid members with multiple chronic conditions	Southern California, United States	Risk score predictor and decision-support for pharmacists offering medicine management to high-risk Medicaid members	General	Monitoring	Risk prediction and decision support	Government
MacPherson et al. (2021)	2021	Adults attending acute primary services	Malawi	Computer-aided digital chest x-ray (DCXR-CAD) for HIV-TB screening	Respiratory	Screening	Automated image analysis	Academia
Mallow and Belk (2021)	2021	Hypothetical cohort undergoing elective orthopedic procedures that commonly have opioids prescribed	United States	Machine learning algorithm analyzing alleles involved in reward pathway of the brain to identify patients with a higher risk of opioid use (OUD)	Analgesics	Diagnostic	Risk prediction	Industry
Mital and Nguyen (2022)	2022	Women aged 40–49	United States	AI to read mammography images to predict breast cancer risk	Oncology	Screening	Automated image analysis	None
Morrison et al. (2022)	2022	Theoretical cohort of infants requiring ROP screening	United States	Artificial intelligence (AI)based retinopathy of prematurity (ROP) screening. Both assistive and autonomous	Ophthalmology	Screening	Automated image analysis	Academia
Nsengiyumva et al. (2021)	2021	Patients with symptoms suggestive of pulmonary TB	Pakistan	AI-based radiograph to triage persons with possible tuberculosis symptoms and identification of those who require further testing	Respiratory	Diagnostic	Automated image analysis	Government
Salcedo et al. (2021)	2021	Adults undergoing active TB treatment	United States	Monitors real-time medication consumption and adherence for TB treatment	Respiratory	Monitoring	Automated image analysis	Government
Schwendicke et al. (2022)	2022	31-year-olds, whose proximal surfaces were initially either good, or in an E2, D1 or D2-3 lesion	Germany	AI-based software to detect proximal caries lesions	Dentistry	Diagnostic	Automated image analysis	None
Szymanski et al. (2022)	2022	Adults aged 65 years or older registered with a GP	United Kingdom	AF risk prediction algorithm to improve AF detection	Cardiology	Screening	Risk prediction	Industry
Tseng et al. (2021)	2021	Hypothetical cohort of asymptomatic 65-year-olds	US	AI ECG algorithm to detect asymptomatic left ventricular dysfunction	Cardiology	Screening	Risk prediction	Academia
Turino et al. (2021)	2021	Adults with newly diagnosed obstructive sleep apnea	Spain	AI monitoring system for improving CPAP compliance	Sleep	Monitoring	Risk prediction and decision support	Government
van Leeuwen et al. (2021)	2021	71,840 adults aged 66 years from a stroke registry that received CTA diagnosis work up of acute stroke	United Kingdom	AI software aiding detection of intracranial LVO in stroke patients	Radiology	Diagnostic	Automated image analysis	None
Xiao et al. (2021)	2021	Asymptomatic adults aged 65 years and above for population screening	China	AI diagnosis of glaucoma	Ophthalmology	Screening	Automated image analysis	Government
Ziegelmayer et al. (2022)	2022	60-year-olds with 20 pack years of smoking history	United States	AI convolutional neural networks supported low dose CT at initial screening for lung cancer	Oncology	Diagnostic	Risk prediction	None

Atrial Fibrillation, AF; artificial intelligence, AI; continuous positive airway pressure, CPAP; CTA, computed tomography angiography; ECG, electrocardiography; European Union, EU; general practice, GP; intensive care unit, ICU; japan society for the promotion of science, JSPS; LVO, large vessel occlusions; Molecular microscope diagnostic system, MMDx; Opioid use disorder, OUD; retinopathy of prematurity, ROP; ROP; tuberculosis, TB.

### 3.3 HEE characteristics

The 21 HEEs contained 10 (10/21, 48%) CUAs, 8 (8/21, 38%) CEAs and 2 (2/21, 10%) BIAs. One (1/21, 5%) HEE reported results as both a CEA and a CUA. Among the CEAs the outcomes ranged from cost saved per patient screened, cost per death averted, cost per DALY averted, cost per case prevented and cost saving per additional tooth retention year. The healthcare system perspective was the most common. Of the 21, 10 (10/21, 48%) took a healthcare system perspective, 6 (6/21, 29%) payer, 4 (4/21, 19%) societal and 1 study (1/21, 5%) took both a societal and health system perspective. In some studies, the payer perspective represented insurers, both public and private.

The time horizon for the 21 studies ranged from 8 weeks to lifetime, with lifetime being the most common (5/21, 24%). One year was the second most common time horizon (3/21, 14%), followed by 6 months and 5 years with two each (2/21, 10%). Time horizons of 8 weeks, 16 months, 3 years, 15 years, 20 years, 30 years, and 35 years were all present in one study each (1/21, 5%). In two studies the time horizon was not reported (2/21, 10%). Most HEEs with a time horizon longer than 1 year used a 3% annual discount rate (7/13, 54%). Six studies discounted costs and health outcomes differentially. Of these, 2 studies (2/13, 15%) discounted costs at 4% and health outcomes at 1.5%, 2 (2/13, 15%) discounted the costs but did not report discount rates for health outcomes, 1 (1/13, 8%) used undiscounted costs but did not report discounting of health outcomes, and 1 (1/13, 8%) did not report discount rates for the costs but discounted health outcomes at 3%. Table 3 reports all the methodological details of the included HEEs.

**TABLE 3:** Health economic details of included studies.

Main author	HEE type	Intervention	Comparator	Perspective	Discount rate	Time horizon	Outcome measure
Adams et al. (2021)	CEA	Combining Artificial Intelligence and Lung-RADS	Lung-RADS	Payer	NA	6 months	Cost saving of AI-informed management per patient screened
Areia et al. (2022)	CEA	AI detection of polyps	Screening without AI tools	Societal	3%	30 years	Cost saving of screening with AI per individual
de Vos et al. (2022)	CUA	AI decision support tool for ICU discharge decision-making	Standard care discharge decisions based on medical expertise	Societal	Costs 4%, Health outcomes 1.5%	1 year	ICER- cost per QALY gained
Delgadillo et al. (2022)	CEA	AI personalized treatment recommendation to provide stratified care	Standard of care- stepped care	Healthcare system	NR	NR	Incremental cost of stratified care per patient and additional case of reliable improvement
Ericson et al. (2022)	CUA/CEA	AI detection of sepsis	Standard care for sepsis diagnosis	Healthcare system	3%	1 year	Cost savings per patient
Fusfeld et al. (2022)	BIA	Pattern recognition in gene expression in biopsy	Histology biopsy alone	Payer	Costs 0%, Health outcomes NR	5 years	Cost per patient and savings per biopsy
Huang et al. (2022)	CUA	AI based DR screening	No screening or ophthalmologist screening	Healthcare system and societal	3%	35 years	ICER- cost per QALY gained
Kessler et al. (2021)	CEA	AI risk score predictor and decision-support for medication management	The same cohort pre-AI intervention start	Payer	NR	NR	Savings per member, per month
MacPherson et al. (2021)	CUA	AI chest x-ray interpretation providing a probabilistic score for TB	Standard of care	Healthcare system	NA	8 weeks	ICER- cost per QALY gained
Mallow and Belk (2021)	CUA	AI prediction to decrease risk of OUD	Current standard of care	Payer	3%	5 years	ICER- cost per QALY gained
Mital and Nguyen (2022)	CUA	Automated mammography image analysis	Alternative screening strategies including no screening, screening guided by risk scores (PRS) and screening guided by family history	Healthcare system	3%	Lifetime	ICER- cost per QALY gained
Morrison et al. (2022)	CUA	Deep learning algorithm	Telemedicine and Ophthalmoscopy	Healthcare system	Costs NR, Health outcomes 3%	Lifetime	ICER- cost per QALY gained
Nsengiyumva et al. (2021)	CEA	AI detection of TB	No AI triage before microbiologic testing. Current standard of care- smear microscopy or GeneXpert	Payer	NA	1 year	Incremental cost per DALY averted
Salcedo et al. (2021)	CUA	AI monitoring for tuberculosis treatment adherence	Standard of care: DOT	Societal	NR	16 months	ICER- cost per QALY gained and NMB
Schwendicke et al. (2022)	CEA	AI detection for proximal caries	Caries detection without AI	Payer	Costs 3%, Health outcomes NR	Lifetime	ICER- cost per year of tooth retention gained
Szymanski et al. (2022)	BIA	AI risk score predictor to detect AF using data from baseline risk factors	Standard care (opportunistic screening and diagnosis) or combined use of standard care and AI	Healthcare system	NR	3 years	Budget impact in £
Tseng et al. (2021)	CUA	AI detection of ALVD	No screening	Healthcare system	3%	Lifetime	ICER- cost per QALY gained
Turino et al. (2021)	CEA	AI monitoring of CPAP compliance	Standard of care	Healthcare system	NA	6 months	Cost per hour of CPAP compliance gained per day
van Leeuwen et al. (2021)	CUA	AI software aiding detection of intracranial large vessel occlusions LVO	Standard of care	Societal	Costs 4%, Health outcomes 1.5%	Lifetime	Incremental cost, incremental effects
Xiao et al. (2021)	CEA	AI detection of glaucoma	No screening	Healthcare system	Costs 5%, Health outcomes NR	15 years	Incremental cost of PACG prevented
Ziegelmayer et al. (2022)	CUA	AI-based CT scan	Stand alone low-dose CT scan	Healthcare system	3%	20 years	ICER- cost per QALY gained

Artificial Intelligence, AI; budget impact assessment, BIA; computerized tomography, CT; cost effectiveness analysis, CEA; cost utility analysis, CUA; diabetic retinopathy, DR; directly observed therapy, DOT; Disability-adjusted life years, DALY; large vessel occlusions, LVO; left ventricular systolic dysfunction, LVSD; net monetary benefit, NMB; opioid use disorder, OUD; Primary angle-closure glaucoma, PACG; reporting and data system, RADS.

### 3.4 Modelling characteristics

Of the 21 HEEs, 16 (16/21, 76%) included a decision analytic model. The modelling characteristics of these are summarized in Table 4. The most common model types were Markov models (6/16, 38%) and decision trees (4/16, 25%) with 3 (3/16, 19%) using a short-term decision tree followed by a longer-term Markov component. Of the remaining 3 studies, there was 1 cost simulation, 1 Markov chain Monte Carlo simulation, and 1 hybrid decision tree and microsimulation model. Authors typically justified their chosen model type by linking the decision to the type of AI intervention, the outcome measure, and the time horizon. Most Markov models used a cycle length of 1 year, and the rest used 1 month or 1 day. Studies that used decision tree models stated their primary reason for doing so was for their simplicity.

**TABLE 4:** Summary of economic evaluation parameters and outcomes.

Main author	Model type	Model states/tree summary	Time horizon, cycle length	Sensitivity analysis	Outcome	Result
Adams et al. (2021)	Cost simulation	NR	6 months	NR	USD 72 to USD 242 saved per patient screened	Intervention cost-effective
Areia et al. (2022)	Markov model	No colorectal neoplasia; low risk adenomas, high risk adenomas, localized, regional, or distant CRC; and CRC-related death	30 years, 1 year	One-way and probabilistic analysis	0.1% absolute (6.9% relative) reduction in colorectal mortality vs. screening without AI, USD 57 saving per individual screened	Intervention cost-effective
de Vos et al. (2022)	Markov model	ICU ineligible, ICU eligible, General ward, Readmission ICU ineligible, Readmission ICU eligible, Discharged, Death	1 year, 1 day	One-way, probabilistic and scenario analysis	EUR 18,507 per QALY gained vs. standard care	Intervention cost-effective
Delgadillo et al. (2022)	Within trial analysis	NR	NR	NR	Incremental cost of stratified care was £104.50 per patient	Intervention potentially cost-effective. Threshold NR
Ericson et al. (2022)	Decision tree	True- and false-positive and true negative detections for sepsis	1 year	One-way and probabilistic analysis	CEA: 356 ICU deaths averted, EUR 2.8m saved/CUA: negative ICER, higher effect, lower cost	Intervention dominant
Fusfeld et al. (2022)	Decision tree	Functioning initial transplant, graft failure + re-transplant, graft failure + dialysis, death with functioning graft, death after graft failure	5 years	One-way and scenario analysis	Savings of USD 19,721 per biopsy over a 5 year period	Produces savings to commercial payers within 2 years
Huang et al. (2022)	Markov model	DR, Mild DR, Moderate DR, VTDR, Stable DR, Blindness and death	35 years, 1 year	One-way and probabilistic analysis	Using health system perspective: USD 1,107.63/QALY vs. no screening, Dominant vs. ophthalmologist screening. Using societal perspective: USD 10,347.12/QALY vs. no screening, Dominant vs. ophthalmologist screening	Intervention cost-effective using both perspectives
Kessler et al. (2021)	Regression analysis	NR	Mean of 20.5 weeks	NR	Saving of USD 554 per member per month	Produces savings
MacPherson et al. (2021)	Within trial analysis	NR	8 weeks	One-way sensitivity analysis	USD 4,520.47 per QALY gained vs. standard of care	Intervention not cost-effective
Mallow and Belk (2021)	Markov chain Monte Carlo simulation model	Alive and Dead. For those who developed OUD: OUD, treatment, remission, dead	5 years, 1 month	One-way, probabilistic and scenario analysis	USD 2,510 saving per patient, 0.02 QALY gain (private insurers), USD 2,682 saving per patient, 0.02 QALY gain (self-insured employers)	Intervention dominant using both perspectives
Mital and Nguyen (2022)	Hybrid decision tree/microsimulation model	No screening, Annual screening for all, AI + no screening for low risk, AI + biennial screening for low risk, PRS + no screening for low risk, PRS + biennial screening for low risk, Family history + no screening for low risk, Family history + biennial screening for low risk. For all interventions any deemed high risk moved to annual screening	Lifetime, 1 year	One-way and probabilistic analysis	AI + no screening for low risk dominated PRS + no screening for low risk, family history + biennial screening for low risk, PRS + biennial screening for low risk, AI + biennial screening for low risk and annual screening for all and extendedly dominated family history + no screening for low risk. USD 23,755 per QALY gained vs. no screening	Intervention cost-effective vs. no screening and dominant vs. other comparators
Morrison et al. (2022)	Decision tree	Ophthalmoscopy, Telemedicine, Assistive AI, Autonomous AI	Lifetime	One-way and probabilistic analysis	Autonomous AI less costly and as effective as telemedicine and ophthalmoscopy. Assistive AI USD 83,350 vs. telemedicine and dominated ophthalmoscopy	Intervention cost-effective
Nsengiyumva et al. (2021)	Decision tree	Triage with AI-based CXR followed by standard of care with upfront smear or GeneXpert	1 year	One-way and scenario analysis	USD 43/DALY averted vs. smear as microbiologic test. Dominant vs. GeneXpert as microbiologic test	Intervention cost-effective
Salcedo et al. (2021)	Markov model	On treatment, Completed treatment, Defaulted	16 months, 1 month	One-way, probabilistic and scenario analysis	AI dominated DOT NMB: USD 3,142, 4,057 and 4,973 at WTP thresholds of USD 50, 100 and 150K respectively	Intervention dominant
Schwendicke et al. (2022)	Markov model	Sound|E1-2|D1|D2-3, True or false negative, No treatment, Development or progression, Restorative Treatment; True or false positive, Treatment, According to dentists’ decision making in each group, Arrested, Restorative treatment	Lifetime, 1 year	One-way sensitivity analysis	AI and no AI showed identical effectiveness and nearly identical costs	Equivalence
Szymanski et al. (2022)	Budget impact model	Opportunistic screening or AI screening, ECG assessment	3 years	One-way and scenario analysis	Standard care + AI generated savings of £71,345,158 and improved clinical outcomes vs. standard care. AI alone generated savings of £80,441,386 but had worse clinical outcomes vs. standard care	Intervention potentially cost-effective. Threshold NR
Tseng et al. (2021)	Decision tree and Markov model	No Screen, Screen with AI algorithm; Treated ALVD, Untreated ALVD, Symptomatic, Untreated no ALVD, Dead	Lifetime, NR	One-way and probabilistic analysis	USD 43,351/QALY vs. no screening	Intervention cost-effective
Turino et al. (2021)	Within trial analysis	NR	6 months	Probabilistic sensitivity analysis	Mean increase of 1.14 h in daily compliance with AI intervention. Non-significant difference in cost between interventions	Intervention cost-effective
van Leeuwen et al. (2021)	Decision tree and Markov model	Patients suspected of stroke receiving CTA, Large vessel occlusion, No or other vessel inclusion; No IAT eligible, IAT eligible; Occlusion detected, Occlusion not detected; mRS 0–5, Death	Lifetime, 1 year	One-way and scenario analysis	AI cost saving of USD 156,000 and gain of 0.01 QALY	Intervention dominant
Xiao et al. (2021)	Markov model	Primary angle closure suspect, primary angle closure, primary angle closure glaucoma, PACG-related unilateral blindness and PACG- related bilateral blindness	15 years, 1 year	One-way sensitivity analysis	USD 1,464 per PACG case prevented over 15 years. Additional healthcare costs from screening were not offset by decreased disease progression over 15 years	Intervention potentially cost-effective. Threshold NR
Ziegelmayer et al. (2022)	Decision tree and Markov model	Decision; CT, CT + AI; Markov; No BC true negative, No BC false positive, BC undetected false negative, BC after resection, BC palliative, Death	20 years, 1 year	One-way and probabilistic analysis	AI CT cost saving USD 67.62 vs. CT screening. AI CT incremental QALY 0.01 vs. CT screening	Intervention dominant

*Self-reported as a simulation model. Artificial Intelligence, AI; asymptomatic left ventricular dysfunction, ALVD; bronchial cancer, BC; chest radiograph, CXR; colorectal cancer, CRC; CTA, computed tomography angiography; Diabetic retinopathy, DR; intensive care unit, ICU; molecular microscope diagnostic system, MMDx; Net monetary benefit, NMB; not applicable, NA; not reported, NR; opioid use disorder, OUD; Primary angle-closure glaucoma, PACG; polygenic risk scores, PRS; standard of care, SOC.

In terms of results, 7 (7/21, 33%) HEEs reported the AI intervention was cost effective versus the comparator relative to an appropriate threshold value, 5 (5/21, 24%) demonstrated that the AI intervention was dominant, and 2 (2/21, 10%) demonstrated equivalence. In 1 (1/21, 5%) study the AI intervention was cost effective versus one comparator and dominant versus the other. In 2 (2/21, 10%) studies the AI interventions produced savings. Three (3/21, 14%) studies did not state a preferred cost-effectiveness threshold to determine if the result was cost effective. The AI intervention was found to be cost ineffective in 1 (1/21, 5%) study.

Of the studies that reported sensitivity analysis (18/21, 86%), 17 reported one-way sensitivity analyses, though the remaining study did conduct probabilistic sensitivity analysis. Seven (7/21, 33%) studies reported both one-way and probabilistic analyses, while 4 (4/21, 19%) reported both one-way and scenario analyses. Three studies (3/21, 14%) reported one-way, probabilistic and scenario analyses.

### 3.5 Quality assessment

A summary of the results from the quality appraisal checklist is shown in Table 5. The assessment resulted in 6 (6/21, 29%) studies with very serious limitations, 11 (11/21, 52%) with potentially serious limitations, and 4 (4/21, 19%) with minor limitations. Initially the two reviewers disagreed on the assessment for two of the studies (Ericson et al., 2022; Mital and Nguyen, 2022). Both were upgraded for the reasons given below.

**TABLE 5:** Summary of quality assessment of included studies.

Study	Notable limitations identified	Assessment
Adams et al. (2021)	Strict assumptions regarding underlying parameters, such as an overestimation of costs, which directly determine the intervention outcome. The 6-month time horizon was short of 12 months deemed best practice by the American College of Radiology, also potentially impacting cost-effectiveness. Finally, the dataset used was not representative of the target populations, notably “overrepresenting white persons and underrepresenting racial minorities"	Very serious limitations
Areia et al. (2022)	Misrepresentation of population data from clinical trials to clinical practice. The overall death rate modelled was lower than the actual. Assumption of compliance of tests and the linear relationship between cancer prevention effect and increased ADR were made, however impact on cost-effectiveness is not severe	Potentially serious limitations
de Vos et al. (2022)	Short time horizon due to literature available for input parameters. Made assumptions from non-Dutch sources which was controlled for with sensitivity analysis, but limits generalisability of results	Potentially serious limitations
Delgadillo et al. (2022)	There were weaknesses regarding the internal validity. The primary outcome was patient reported, and used a general measure rather than disorder specific measures. The majority of patients were white which has generalizability implications	Potentially serious limitations
Ericson et al. (2022)	Limitations arise from patients who should have been included for Sepsis, not included. The model base case was purposely set to be conservative to not exaggerate the positive effects, however the assumptions made limits the validity of the outcomes. Finally, the research and funding were funded by the company who developed the intervention, creating potential for bias	Very serious limitations
Fusfeld et al. (2022)	The model does not capture adverse events due to antirejection medication which they suspect MMDx would increase leading to uncertainty in the result. There is also a potential conflict of interest where the research was funded by the company which developed the AI technology	Very serious limitations
Huang et al. (2022)	Limited data available from study population led to values derived from other countries which were accounted for in sensitivity analysis. Data regarding sensitivity and specificity of the AI screening derived from one paper, but did not greatly affect cost effectiveness in the sensitivity analyses	Minor limitations
Kessler et al. (2021)	Retrospective observational study limits conclusions on causality. Clinical outcomes were not analyzed	Potentially serious limitations
MacPherson et al. (2021)	Trial-based analysis with small number of events and short follow up resulted in less precise treatment estimates. Study presence in the clinic may have modified health worker behaviour for standard of care. Alternative diagnoses to TB were not investigated	Minor limitations
Mallow and Belk (2021)	The model assumed all patients would consent to the test which excludes the costs and effects if patients refused. The model also did not exhaust all features of the treatment pathways due to the high number of possibilities	Potentially serious limitations
Mital and Nguyen (2022)	Main limitation is the cost of using AI for breast cancer prediction is not yet known in clinical practice which led to data retrieved from the European Society of Radiology. This was accounted for with one-way sensitivity analysis with all results holding. Data for efficacy of AI intervention extrapolated beyond studied period	Potentially serious limitations
Morrison et al. (2022)	Speculative assumptions and imprecision in model inputs. However, the authors used conservative estimates and performed sensitivity analyses. Model time horizon was lifetime despite the life expectancy in the population (very premature babies) being unknown	Potentially serious limitations
Nsengiyumva et al. (2021)	The analysis examines the intervention in low HIV prevalence, the accuracy of results may vary in high prevalence	Minor limitations
Salcedo et al. (2021)	The model did not consider possible side effects or delays in appropriate care due to less nurse contact. Relatively short time horizon that assumes equal quality of life post-treatment between arms	Potentially serious limitations
Schwendicke et al. (2022)	Range of sources for input data which will lead to a degree of bias, although accounted for in sensitivity analyses. Lacked validity as in practice treatment decision would not be based on image analysis only	Potentially serious limitations
Szymanski et al. (2022)	Used an unvalidated threshold to determine AF risk and assumed 100% adherence to ECG assessment which lacks external validity. Did not include cost of implementation. The study was funded by the AI developer	Very serious limitations
Tseng et al. (2021)	The data estimates for the baseline (SOLVD) probabilities and effects were based on a study published 30 years ago from the last RCT. The model was calibrated to use a prespecified threshold which was not varied in the sensitivity analyses. There is also a conflict of interest where the research was funded by the organization which developed the AI technology	Very serious limitations
Turino et al. (2021)	Patients with severe chronic pathologies were excluded which could limit the generalizability of results and the follow-up period is relatively short. The study collected EQ-5D data but did not report utility data	Potentially serious limitations
van Leeuwen et al. (2021)	Model relied on two key inputs that were assumptions: percentage of missed LVOs in practice, and the capability of the AI to reduce missed LVOs. These were both varied in the sensitivity analyses and result did not change. The model only included early presenters but IAT would also include late presenters which limits generalizability. The authors also assumed that false positives would be neutralized by the reader and would not lead to unnecessary care	Minor limitations
Xiao et al. (2021)	The predictive accuracy of the intervention came from the literature and may not be generalizable to the setting. Any varying of this was not reported. There was a lack of robust data on the efficacy of treatment that followed a positive screening result which was accounted for in the sensitivity analysis	Potentially serious limitations
Ziegelmayer et al. (2022)	Input parameters came from multiple sources including assumptions and numerous published studies, leading to a degree of bias. Varying the specificity of the AI or CT and cost of AI greatly increased the ICER changing the result from intervention dominant to not cost-effective	Very serious limitations

Studies deemed to have very serious limitations were those where an issue in 1 or more quality criteria were highly likely to materially change the cost-effectiveness conclusion for the AI intervention. There were several key reasons which led to this assessment for 5 of the included studies. In one there was an acknowledged overestimation of cost data, representation issues between the dataset and target population, and a short 6-month horizon rather than the 12-month time horizon deemed best practice by the American College of Radiology (Rosenthal and Dudley, 2007). In another, adverse health effects were not captured, which the authors suggested would increase the cost-effectiveness estimate (Fusfeld et al., 2022). This study also had a financial conflict of interest where research was funded by the company which developed the AI intervention. This was true for another 2 studies (Ericson et al., 2022; Szymanski et al., 2022). In another study, the result changed from intervention dominant to cost ineffective when input data, arising from multiple sources and assumption, were varied during the sensitivity analyses (Ziegelmayer et al., 2022).

Studies with potentially serious limitations tended to have a paucity of appropriate input data. Instead, alternative sources, or multiple sources were used with resulting generalizability issues. It was common for studies to have assumptions for the cost and effectiveness of the AI intervention, compliance, and the impact of the AI intervention on the subsequent treatment pathway. Examples of this are 1 study that assumed all patients would consent to a test (Mallow and Belk, 2021); 1 study that used a primary outcome that was patient reported (Delgadillo et al., 2022) and 1 study that assumed the effectiveness of the AI intervention last for 10 years, despite having data for only 5 years (Mital and Nguyen, 2022). These studies did account for the key uncertainties in sensitivity analyses and the effect was either minor or the initial assumptions were shown to be robust. Some studies were assessed as having potentially serious limitations due to unclear reporting, which reduced transparency around key information such as whether a cost had been applied for the AI intervention, how it would integrate with clinical care, and who the anticipated user of the AI intervention was.

## 4 Discussion

This paper systematically reviewed 21 HEEs of AI interventions. The studies mainly evaluated AI-based automated image analysis interventions for diagnosis and screening in general medicine, oncology and ophthalmology. Nearly all were CUAs and CEAs that took a healthcare system or payer perspective, and most had lifetime time horizons. Some of the HEEs were trial-based analyses, but the large majority were model-based which mostly used Markov models. In terms of the HEE results, the AI interventions were cost effective or dominant in just over half and all the studies performed sensitivity analyses.

This study reports an updated search to the review conducted by Voets et al. (Voets et al., 2022), providing a contemporary snapshot of the HEE evidence base for AI health technologies Our update captures an additional 15-month period in a time where AI health based technologies are on the exponential rise, evidenced by the near quadruple number of initial unique search results since April 2021 (Voets et al., 2022). It appears there has been no change in the most commonly evaluated purpose of AI being used as a healthcare intervention, as Voets et al. also found the most common to be automated image analysis (Voets et al., 2022). Ophthalmology and screening were the dominant specialty and phase of the care pathway at which the AI intervention was used, and these were also prevalent in this updated review. The prevailing type of HEE in the original review was cost minimization with the preferred outcome measure of cost saved per case identified. This was common among our included studies, although we termed it CEA, but CUA was the most common study type in this update. There was a difference between the two reviews in how many of the technologies were found to be cost saving. Voets et al. found the majority were whilst this was true for only 2 studies in this review. This could be due to differences in applying the terms ‘cost-saving’ and ‘cost-effective’ as a large proportion of studies in this updated review were cost-effective.

Another difference was the fact that the large majority of HEEs in our review were model-based, compared to 45% of those in Voets (Voets et al., 2022). This could suggest a shift towards using models to estimate future costs and benefits of AI technologies, permitting longer time horizons than trial-based evaluations (the most common time horizon is our review was lifetime, compared to 1 year in Voets). Furthermore, the increasing use of model-based evaluations may suggest AI interventions are moving towards traditional value assessment frameworks that are commonplace in the health technology assessment of medicines. This increase in model-based technologies may also explain the differences in results regarding cost saving versus cost effective. Perhaps it is easier or more expected to generate cost-effectiveness estimates when using a model compared to non-model HEEs where it may be more common to focus on costs.

Voets et al. (2022) found that the evidence supporting the chosen analytical methods, assessment of uncertainty, and model structures was underreported. Our quality assessment determined that most studies had potentially serious limitations tending to arise from the sources and assumptions regarding the input data. These findings are consistent, which suggests that despite an increase in the use of more sophisticated economic evaluation techniques, the evidence supporting them remains limited. In some cases, the uncertainty and lack of clarity for the reader were due to the reporting of the HEE rather than the data quality. In numerous studies it was hard to determine fundamentals such as whether a cost had been applied for the AI intervention, how it would integrate with clinical care and who the anticipated user of the AI intervention was. As mentioned, not all of the studies we identified clearly stated how the AI intervention would integrate with clinical care. Studies did not typically thoroughly or transparently estimate subsequent care and downstream health outcomes resulting from the use of an AI intervention. Our findings from this literature review suggest this is an area that needs to be better considered and reported.

AI-based interventions have the potential to be distinct from traditional medical interventions if they can learn (from data) over time. Theoretically, this means the relationship between the intervention and outcome may not be fixed; an AI intervention could get *more* effective over time, unlike the typical effect *waning* assumption associated with medicines. This has implications when considering future benefits and how to extrapolate this over the time horizon of the HEE. The prevailing model structures used in HEEs of AI interventions to date—Markov models, decision trees, and hybrids of the 2—may limit the extent to which studies have been able to capture and examine the dynamic nature of AI interventions. Therefore, there is the possibility that the existing HEE evidence base has not captured the true potential value of many AI interventions due to limitations imposed by their model structures, and only a third of our included studies explored the impact of structural uncertainty in sensitivity analysis. Furthermore, traditional, ‘simple’ models may not facilitate easy modelling of downstream costs and benefits, by quickly becoming slow or unwieldy. This, potentially, fails to show the full benefit of the AI intervention, inhibiting implementation. Guo et al. (Guo et al., 2020) acknowledge this through a paradox of “no evidence, no implementation—no implementation, no evidence”. More sophisticated types of model, that are less restricted by the structural limitations that affect simple decision tree and Markov models may be better placed to capture full pathway effects in addition to potential time-dependent effectiveness of AI-based interventions.

Simulation-based modelling presents the opportunity to build flexible, sophisticated models that can overcome several limitations of Markov models and decision trees. They can easily incorporate the history of past events, model factors that can vary between patients and have a non-linear relationship with outcomes, and do not use discrete time intervals (Davis et al., 2014). They can also track the path of each person over time and estimate individual-level effects or mean group-level effects for a population (Davis et al., 2014). These possibilities may lead to models capable of addressing the potential dynamic nature of AI interventions learning over time and the impact on linked decision points and subsequent care in a clinical pathway. As data on AI-based interventions continues to be collected and reported, the ability to develop these models should improve. One thing to note, however, is that for these models to underpin reimbursement decisions HTA agencies would need to be able to critique and utilize them. This may require new skills, knowledge and experience and present other challenges. Utilizing these sorts of models also leads to the debate of whether HTA should be more ‘living’. This refers to regular and scheduled updates of recommendations instead of the more traditional ‘one-off’ decisions. Living HTA presents opportunities as well as challenges (Thokala et al., 2023) and is not yet common practice.

The usefulness of a published HEE for decision making depends on how well it is conducted and reported. Reporting guidelines play an important role in improving transparency and completeness and as new technologies emerge, can help drive best practice. A prominent reporting standard within the field of HEEs is the Consolidated Health Economic Evaluation Reporting Standards (CHEERS) (Husereau et al., 2022). This outlines minimum reporting standards and was recently updated in 2022. It includes a 28-item checklist covering methodological approach, data identification, model inputs, assumptions, uncertainty analysis, and conflicts of interest. It does not include any reporting items that are specific to any AI components of the intervention, but the authors did recognize that CHEERS could be more specific for certain situations and welcomed opportunities to create additional reporting guidance. An extension to CHEERS covering AI specific items could improve the reporting, transparency and ultimately decision making for AI interventions. This could also help mitigate the paradox of poor reporting inhibiting adoption of AI interventions.

The system-wide need and motivation for improving best practice around data collection and transparency for AI health interventions is evident. Extensions for AI technologies have already been developed for other checklists. CONSORT-AI (Liu et al., 2020) contains AI-specific items for the reporting of RCTs, and it was done in collaboration with the SPIRIT-AI extension for trial protocols (Rivera et al., 2020). Including AI-specific items in the reporting of HEEs may be a logical step to contribute to this standard setting and help to ensure that all relevant information is available to decision makers.

### 4.1 Limitations

This study has some limitations. We updated the Voets et al. systematic literature review, but searched different databases. It is possible there may have been relevant studies within our search window that we missed by not searching the same databases; however, we believe the databases we searched should give at least equivalent, and probably superior, sensitivity to the original review. Indeed, the sensitivity of our search strategy is evidenced by the large number of studies excluded at primary screening (2,993) relative to the total number of unique records (3,033). The sensitivity of HEE search filters is well known (Hubbard et al., 2022). While this means our review is highly likely to have identified all relevant published studies, it does mean further updates may be labor intensive with lots of records to screen to identify a relatively small number of relevant studies.

Our review specifically focused on economic evaluations and whilst out of scope, some studies, such as those only reporting patient reported outcome measures, may have been of interest to readers. Additionally, a potential limitation is that our search only covered the period from 1 April 2021 to 17 June 2022. This relatively short search period remains informative due to the rapid advent of AI in healthcare, but it also means that it is likely that relevant economic evaluations have been published since our review.

Another limitation relates to the subjective nature of the NICE quality appraisal checklist. Although the checklist allowed for a further level of analysis regarding the quality of the economic evaluation, it should be used as a broad interpretation rather than a critique of any given study. Despite negating any potential bias by having 2 reviewers, it is possible that different reviewers may have implemented the checklist differently and produced different results. Additionally, other, similar checklists exist (Philips et al., 2004; Drummond, 2015; Adarkwah et al., 2016), and although they broadly serve a similar purpose of understanding the methodological limitations of HEEs, they may have resulted in different or more nuanced quality assessments.

## 5 Conclusion

This updated review, while covering just a 15-month window, found more economic evaluations of AI health interventions since the last comprehensive systematic literature review which covered the preceding 5 years. Many of the included studies were model-based evaluations and the most common AI intervention was automated image analysis used for screening or diagnosis in the areas of general medicine and oncology. Most evaluations reported the cost per QALY gained.

Overall, the reporting of the studies exhibited limitations. Only a small number of studies were judged to have just minor limitations, according to application of the NICE quality assessment checklist. The majority had potentially serious or very serious limitations resulting from conflicts between research funding and authorship, uncertainty in input data changing the outcome of the evaluation, and lack of transparent reporting of key elements, such as the cost of the technology and how it will be implemented into clinical practice. Specific reporting standards for the economic evaluation of AI interventions would help to improve transparency, reproducibility and trust, and promote their usefulness for decision making. This is fundamental for implementation and coverage decisions which in turn will generate the necessary data to develop flexible models better suited to capture the potentially dynamic nature of the AI intervention.

## Data Availability

The raw data supporting the conclusion of this article will be made available by the authors, without undue reservation.
