# Supplementary figures and images for: Economic evaluations of artificial intelligence-based healthcare interventions: a systematic literature review of best practices in their conduct and reporting
Source: Front Pharmacol. 2023 Aug 8;14:1220950. doi: 10.3389/fphar.2023.1220950 (PMC10486896; doi:10.3389/fphar.2023.1220950)

#
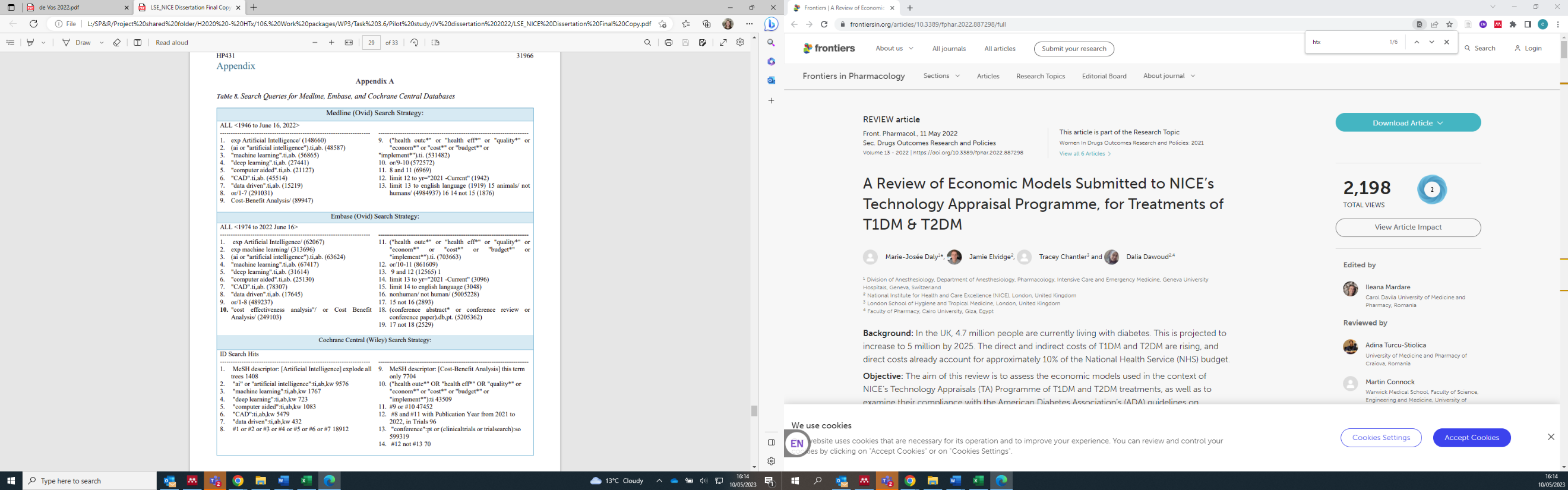
Appendix A

Supplement: Supplementary file 1 [file Table1.DOCX]
